# Production of Biosurfactants by Soil Fungi Isolated from the Amazon Forest

**DOI:** 10.1155/2018/5684261

**Published:** 2018-04-24

**Authors:** Hellen Holanda Sena, Michele Alves Sanches, Diego Fernando Silva Rocha, Walter Oliva Pinto Filho Segundo, Érica Simplício de Souza, João Vicente Braga de Souza

**Affiliations:** ^1^Post-Graduate Program in Pharmaceutical Sciences, Federal University of Amazonas (UFAM), R. Alexandre Amorim 330, 69010-300 Manaus, AM, Brazil; ^2^Department of Medical Microbiology, National Institute of Amazonian Research (INPA), Av. André Araújo 2936, 69080-971 Manaus, AM, Brazil; ^3^Superior School of Technology, Amazonas State University (UEA), Av. Darcy Vargas 1200, 69050-020 Manaus, AM, Brazil

## Abstract

Biosurfactants are surface-active compounds that have sparked interest in recent years because of their environmental advantages over conventional surfactants. The aim of this study was to investigate the production of biosurfactants by soil fungi isolated from the Amazon forest. Fungi colonies were isolated from soil samples and screened for biosurfactant production in submerged fermentation. In addition, the influences of bioprocess factors (carbon source, nitrogen source, pH, and fermentation time) were investigated. Finally, the biosurfactant produced was semipurified and submitted to stability tests. One hundred fungal cultures were obtained from the soil samples, identified by micromorphology, and submitted to screening for biosurfactant production. Sixty-one strains produced biosurfactants. The strain* Penicillium *8CC2 showed the highest emulsification index (54.2%). The optimized bioprocess conditions for biosurfactant production by* Penicillium* 8CC2 were as follows: soybean oil, 20 g/L; yeast extract, 30 g/L; pH 9; duration of 9 days. The semipurified biosurfactant showed stability after heating at 100°C for 60 min and after the addition of 30% NaCl (w/v). Tween 80 (0.2% w/v), a conventional surfactant, was used as the control.

## 1. Introduction

Surfactants are among the most versatile materials in chemical and process industry. Its amphiphilic nature, containing both hydrophilic and lipophilic functional groups in one molecule, plays an important role in numerous chemical applications (dispersion systems, as emulsions and colloids, personal hygiene, detergents, fabric softeners, emulsions, and paints over food texture) [1, 2]. Recently, literature has been demonstrating the advantages of biosurfactants (surfactant of biological origin) if compared with surfactants from chemical industry: lowest toxicity, higher biodegradability, and possible biological activities [2, 3]. Biosurfactants are a structurally diverse group of compounds produced by organisms that have surface-active properties and emulsification action. Biosurfactant properties are similar to the surfactant; they have industrial applications in relation to detergency, emulsification, lubrication, foaming capacity, ability, solubility, dispersion phases [2–5], and another interesting application in catalysis, biosensing, and electronics using microstructures [6].

Biosurfactants are mainly produced by bacteria and yeast, but, in recent years, studies have highlighted their production by filamentous fungi as well [7, 8]. These microorganisms are potential producers of various substances of biotechnological interest, such as pigment enzymes and antibiotics, and their main sources of isolation are plants and soil [9]. The production of biosurfactants by microorganisms is influenced by several factors, including the nature of the carbon source and the concentrations of nutrients such as nitrogen, phosphorus, magnesium, iron, sulfur, and manganese, as well as the pH, temperature, agitation, and available oxygen [2, 10, 11]. These factors can make the production of biosurfactants more expensive than that of synthetic surfactants [12]. Moreover, several studies have been performed to make the price of the bioprocess more competitive [13].

Despite intensive efforts to study the Amazon, we lack knowledge of the microbial diversity and the production of substances of biotechnological interest by the filamentous fungi that make up this biome. Amazonian bioprospecting studies such as this one can result in the discovery of fungi with high productivity and new biosurfactants. In the current study, we investigated the production of biosurfactants by fungi isolated from soil samples of the Amazonian forest. Analyses were performed related to the following: (i) isolation and identification of biosurfactant-producing fungi, (ii) optimization and kinetic parameters related to the production of the biosurfactant selected, and (iii) comparison of the emulsion stability of the biosurfactant of fungal origin compared to that of a conventional surfactant.

## 2. Materials and Methods

### 2.1. Isolation and Identification of Soil Fungi

The samples were collected at six locations in the woods of the National Institute of Amazonian Research (INPA) (3.10′39′′S; 59.96′′77′′W), Amazonas, Brazil. For isolation, 1 g of soil was transferred to a tube containing 9 mL of water, and a 1 : 100 dilution was prepared. Then, 100 *μ*L of the diluted sample was plated on a Petri dish containing Sabouraud agar with chloramphenicol (250 mg L^−1^). After 72 h of incubation at 25°C, the colonies that developed were transferred to tubes with the same medium; the test was performed in triplicate. A streaking technique was performed to obtain monosporic crops. Fungal colonies were identified by observing macroscopic aspects of the colonies and microscopic features, as described by Lacaz et al. [14] and Barnett and Hunter [15].

### 2.2. Selection of Biosurfactant Producers

The selection of biosurfactant producers was performed in Erlenmeyer flasks (125 mL) containing 25 mL of culture medium (with 40 g L^−1^ soybean and 20 g L^−1^ peptone) as previously described by Accorsini et al., 2012; however, the mineral solution was replaced with peptone [16]. The medium was inoculated with spores of the isolates at a final concentration of 1 × 10^4^ spores/mL^−1^. Then, the flasks were incubated without shaking at room temperature (25 ± 2°C) for 7 days, followed by filtration with a quantitative filter paper (Ø, 12.5 cm; pore size, 28 *μ*m; Quanty®) for removal of fungal cells. The filtrate was separated for use in detection of biosurfactants by the drop collapse method described by Bodour and Miller-Maier [17] and the emulsification index described by Cameron et al. [18].

In the drop collapse test, a polystyrene plate with 96 microwells (8.5 × 12.7 cm) was used. The plate was washed with hot water, ethanol, and distilled water. Then, 5 *μ*L of the filtrate of each sample was inoculated separately into wells prefilled with 2.0 *μ*L of mineral oil previously left at room temperature for 24 h. After 1 min of the reaction, the result was determined visually. The result was considered positive when the drop of mineral oil collapsed. A control was prepared using SDS at a concentration of 25%.

To test the emulsification index (E24), a 4 mL aliquot of the culture filtrate (cell-free) was mixed with 6 mL of toluene in a screw tube. The mixture was shaken vigorously for 2 min on a tube shaker-type vortex (Phoenix®). After 24 h, the ratio of emulsified toluene was compared with the total volume. The emulsification index was calculated using the following formula:

(1) $$\begin{array}{c} E24=\left(\;\frac{\text{height of the emulsion layer}}{\text{total height}}\;\right)\times 100. \end{array}$$

### 2.3. Influence of Carbon and Nitrogen Sources on Biosurfactant Production

The influences of different carbon (20 g L^−1^) and nitrogen (10 g L^−1^) sources on biosurfactant production were evaluated. The experimental conditions were the same as those described in the Selection of Biosurfactant Producers, and the results for each substrate were analyzed using univariate analysis. The carbon sources investigated were soybean oil, starch, sucrose, cellulose, and xylose, and the nitrogen sources investigated were peptone, yeast extract, meat extract, sodium nitrate, and malt.

### 2.4. Effects of Concentration of Carbon and Nitrogen Sources, pH, and Treatment Time

We evaluated the influence of soybean oil, yeast extract, pH, and bioprocess time through a 2^*k*^ factorial design (two levels). The chosen design was 2^4^ with four central points (evaluation of experimental error), as described by Neto et al. [19]. The other experimental conditions were similar to those described in the Selection of Biosurfactant Producers.

### 2.5. Semipurification of Biosurfactant and Stability Analyses

Semipurification of the biosurfactant was performed with 30 repetitions under optimized conditions. The resulting solutions were pooled, filtered, and precipitated with ethanol (1 : 4 v/v, 4°C, 48 h). The mixture was subjected to centrifugation (5000 rev/min for 20 min), and the precipitate obtained was used for stability testing.

The effect of the addition of NaCl (30% w/v) was evaluated. After the addition of salt to the 1% w/v solution of the precipitate containing the biosurfactant, the emulsifying activity was tested using the emulsification index.

The effect of temperature on the biosurfactant activity was investigated by keeping 1% w/v of the precipitate containing the biosurfactant at 100°C in a water bath for 60 min and verifying the emulsification index. The synthetic surfactant Tween 80 (1% w/v) was used as a control substance in these assays.

### 2.6. Statistical Analysis

Statistical analyses were performed using STATISTICA versions 5.0 and 6.0 (STATGRAPHICS, Statpoint Technologies, Inc., Warrenton, VA, USA). All experiments were performed in triplicate for the calculation of mean and standard deviation.

## 3. Results

### 3.1. Isolation and Identification of Fungi

One hundred fungal strains were screened for their biosurfactant production. We obtained colonies from the genera* Penicillium* (46 isolates),* Aspergillus* (24 isolates),* Fonsecaea, Gliocladium* (one isolate),* Trichoderma* (12 isolates),* Fusarium* (eight isolates), and* Acremonium* (two isolates). Additionally, from the phylum Zygomycota, colonies from the genus* Mucor* (six isolates) of the class Zygomycetes were obtained.

### 3.2. Selection of Fungal Producers of Biosurfactants

To select the biosurfactant-producing fungi, we performed analyses of bioprocessing in submerged fermentation and evaluated the emulsification index and drop collapse test (Table 1) of the culture filtrate. Nine isolates showed a high emulsification index (E24 > 40); these belonged to the genera* Penicillium* (three isolates),* Trichoderma* (three isolates), and* Fusarium* (three isolates). The isolated* Penicillium* 8CC2 showed a high emulsification index (54.2%) and was selected for the remaining stages of the study because its emulsion could be maintained for more than 15 days; others have isolated emulsification rates below 30%.

### 3.3. Effect of Carbon and Nitrogen Sources on Biosurfactant Production

**Figure 1:**
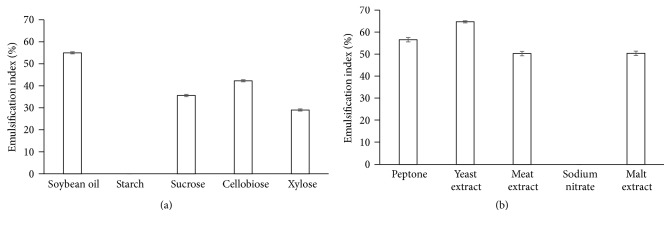
Univariate test measuring the influence of carbon and nitrogen sources in the production of biosurfactants by* Penicillium *8CC2. (a) Carbon source and (b) nitrogen source.

To optimize the production of biosurfactants by the isolated* Penicillium *8CC2, we investigated the influence of carbon and nitrogen sources by performing univariate experiments (Figure 1). The carbon and nitrogen sources that enabled the highest production rates were soybean oil (55%) and yeast extract (64,7%), respectively.

### 3.4. Influence of Soybean Oil, Yeast Extract, pH, and Time on Biosurfactant Production

The influence of soybean oil and yeast extract concentrations, pH, and time on biosurfactant production by* Penicillium *8CC2 was investigated using the experimental design 2^4^, as described with four replicates added at the central point (Table 2). All major factors and some interactions (yeast extract × time and pH × time) showed statistical significance (Table 3). An analysis of variance (ANOVA) was performed to validate the mathematical model presented (see (2)); the related information is provided in Table 4. The mathematical model showed a significant regression (91.8%), and its factors showed statistical significance. The lack of fit was not significant (*p* > 0.05).

(2) E24=8,1375−0,128125×Soybean  Oilg/L−0,534375×Yeast  Extractg/L+10,4063∗pH+4,5∗Timeh+0,184375×Yeast  Extractg/L×Timeh−1,15625∗pH∗Timeh.

**Figure 2:**
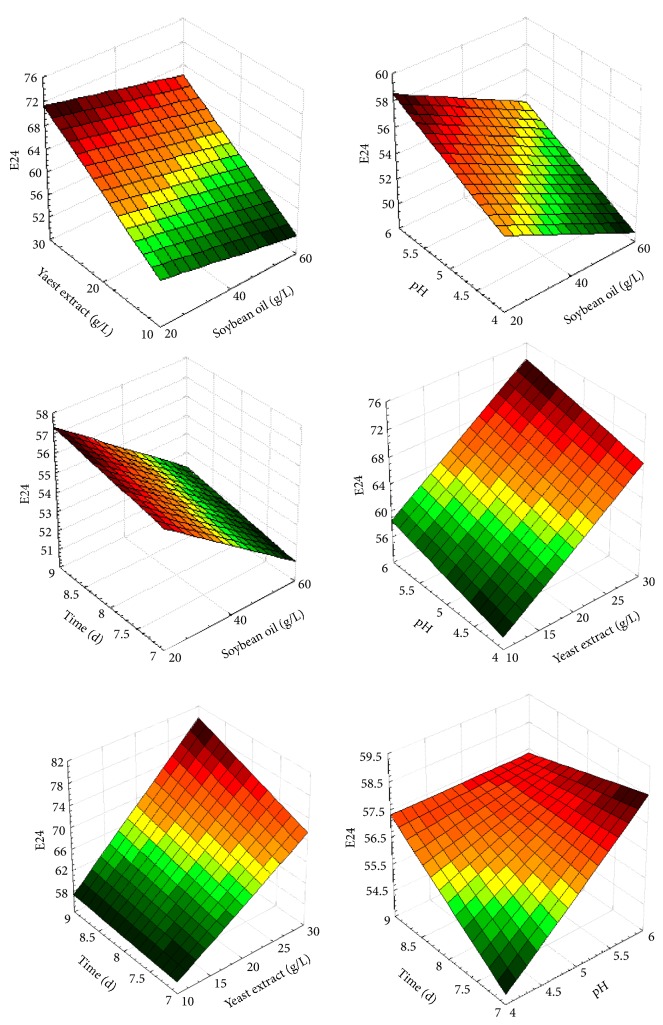
Response surface analysis for biosurfactant production by* Penicillium* 8CC2, with soybean oil, yeast extract, pH, and production time.

To represent the estimated response (E24), surfaces were prepared (Figure 2) based on the data generated by the model. The surfaces constructed on the basis of the model responses showed that the optimal conditions for maximal biosurfactant production were as follows: soybean oil, 20 g L^−1^; yeast extract, 30 g L^−1^; pH 6; and a duration of 9 days. These values were obtained with a theoretical E24 of 79.82%.

### 3.5. Stability Studies

**Figure 3:**
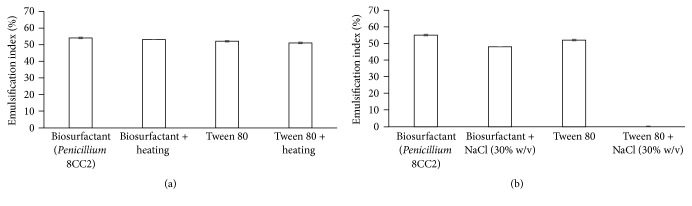
Stability of biosurfactants from* Penicillium *8CC2 under different physical conditions: (a) after heating 100°C (60 min) and (b) with the addition of NaCl (30% w/v). The synthetic surfactant Tween 80 (1% w/v) was used as a control substance in these assays.

The stability of the new biosurfactant was evaluated under different physical conditions (Figure 3). Heating (100°C for 60 min) did not influence the biosurfactant activity. A high NaCl concentration caused a reduction of 12.7% in the E24 value of the biosurfactant produced by* Penicillium* 8CC2 and completely inhibited the emulsion formation by the commercial surfactant Tween 80.

## 4. Discussion

We found* Penicillium* 8CC2 (strain isolated from Amazonian soil) produced biosurfactant with high emulsification index (EI24 > 50). In addition, we found that the biosurfactant from* Penicillium* 8CC2 has similar thermal-stability and higher NaCl-stability if compared to the surfactant Tween 80. The present work was the first to screen the potential of fungus obtained from soil samples of the Amazon forest to produce biosurfactant and the selected microorganisms and their biosurfactant have industrial potential.

Among the fungi obtained from soil, the genera that showed a strong ability to emulsify toluene were* Penicillium*,* Trichoderma*, and* Fusarium*. The potential of* Penicillium* sp. to produce biosurfactants has already been demonstrated [20, 21], but few studies have described the biosurfactant potentials of* Trichoderma* [22] and* Fusarium*.

Carbon sources for microbial production of biosurfactant can be obtained from carbohydrates, vegetable oils, hydrocarbons, and waste frying oils [23, 24]. In this study, soybean oil was the best carbon source for biosurfactant production. Soybean oil was also described as a suitable carbon source for fungal production of biosurfactant by Accorsini et al. [16]; these last ones investigated more affordable culture medium to produce biosurfactant by yeasts. We believe that triglycerides composition is a natural inducer of the biosurfactant production, since the microorganisms produce biosurfactant to increase the access to lipid degradation and it is clear that the triglycerides can be directly used to anabolize the production of the surfactant. Starch, despite being used by other microorganisms as a carbon source [25], does not appear to be a suitable substrate for biosurfactant production by* Penicillium* 8CC2.

Biosurfactants are produced when there is nitrogen limitation during the stationary phase of growth of biomass [26–28]. In the present work, yeast extract was found to be the most suitable nutrient source for the production of the metabolite of interest, followed by peptone, meat extract, and malt. The mineral nitrogen source sodium nitrate was not suitable for production of the biosurfactant. Another study showed similar results; it was observed that* Torulopsis bombicola* produced biosurfactants using yeast extract but was not able to produce them using sodium nitrate.

The results of this study showed that the factors studied (soybean oil, peptone, pH, and time) and some of their interactions had a significant effect on the response variable (biosurfactant production). The validated mathematical model demonstrated that an E24 of 79.82 could be obtained using 20 g L^−1^ soybean oil, 30 g L^−1^ yeast extract, pH 6, and a 9-day duration of the bioprocess. Maximum biosurfactant production occurred in the optimal pH range employed for microorganism growth. During the production of this biosurfactant, as well as during any chemical reaction, the pH directly affects the microbial activity because of the effects of H^+^ ions on cell permeability and enzyme activity [29]. Kiran et al. [30] reported that an* Aspergillus ustus* isolate showed optimal biosurfactant production at pH 7.* Mucor circinelloides* was found to maximally produce biosurfactant at pH 8 using apple peel, vegetable oil, and corn water as substrates [31]. The time required for maximum biosurfactant production by* Penicillium* sp. 8CC2 in the current study was 9 days.* Aspergillus fumigatus* was found to produce biosurfactant in 112 h in solid-state fermentation [32].* Aspergillus ustus* isolated from a marine sponge showed optimized production at 120 h in Sabouraud dextrose broth [30].

The stability of the biosurfactant was evaluated at high temperatures and high ionic strengths. After subjecting the biosurfactant to a temperature of 100°C for 60 min, there was no verified change in biosurfactant activity. This result demonstrates the potential utility of the biosurfactant in the food, pharmaceutical, and cosmetic industries where heat sterility is of great importance [32, 33]. In the case of ionic strength, there was a small reduction in the emulsification ability of* Penicillium* 8CC2 when NaCl (30%) was added, but a stable emulsion could still be maintained. The commercial emulsifier Tween 80, used as a control, did not remain stable when NaCl was added. This result demonstrates that the biosurfactant produced by* Penicillium* 8CC2 can be used in high salt-concentration conditions, for example, in tertiary oil recovery activities.

This study verified stability parameters for only one fungus from the 61 potential biosurfactant-producing fungi, thus ignoring possible potentiality in many others, including some with high levels of emulsification. In the stability tests, we could have assessed the influence of pH. The results demonstrate that biotechnological approaches can be used to improve biosurfactant production by* Penicillium* 8CC2. The current results suggest the potential usefulness of the biosurfactant produced by a filamentous fungus isolated from soil samples from the Amazon region.

The data from the present work is important since a significative number of isolates (100 cultures isolated from soil samples from the Amazon) were investigated for the production of biosurfactant. The analytical methodology used in the screening of biosurfactant producers did not quantify the biosurfactant directly in the culture filtrate; nowadays, the screening methods have been developed that rely on the interfacial activity of the biosurfactants but do not measure it directly. We used methodology that quantifies the ability of the biosurfactant present in the culture filtrate to produce emulsification with toluene “emulsification index” as classically described by Mullingan et al. [34]. Unfortunately, as a work limitation, during the experimental conditions, it was not possible to identify the species of* Penicillium* 8CC2 and the chemical composition of the biosurfactant. Literature does not have description of chemical composition of the biosurfactants produced by* Penicillium *sp. and further works are necessary to elucidate it.

The development for screening microbes from thousands of potentially active organisms and the subsequent evaluation of surface activity holds the key to the discovery of new biosurfactants or production strains.

## Figures and Tables

**Table 1:** Fungal cultures isolated from soil samples and results for the best producers of biosurfactants, obtained using the drop collapse test and emulsification index (E24).

Isolated organism	Drop collapse test	Emulsification index
*Fusarium *sp. 87LIV12	Neg	64.28
*Fusarium *sp. 86LV272	Neg	58.57
*Fusarium *sp. 85RV342	Neg	57.14
*Penicillium* sp. 97VV74	Neg	57.14
*Penicillium* sp. 8CC2	Neg	54.2
*Trichoderma* sp. 91BN312	Neg	52.85
*Trichoderma* sp. 60AVR3	Pos	51
*Trichoderma* sp. 16AC2	Neg	42.85
*Penicillium* sp. 62BLC2	Neg	40.3

**Table 2:** Influence of soybean oil, yeast extract, pH, and time on biosurfactant production by *Penicillium* 8CC2, performed according to a factorial design.

Soybean oil (g l^−1^)	Yeast extract (g l^−1^)	pH	Time (d)	E24
40	20	5	7	61
60	30	4	9	76
60	10	4	9	53
40	20	5	7	60
60	30	6	9	76
20	30	6	9	80
20	10	4	5	47
40	20	5	7	58
40	20	5	7	62
60	30	4	5	51
20	10	6	9	62
20	10	6	5	56
60	30	6	5	60
20	30	6	5	69
60	10	4	5	48
20	10	4	9	58
60	10	6	5	60
20	30	4	5	62
20	30	4	9	78
60	10	6	9	47

**Table 3:** Effect of tested variables on biosurfactant production by *Penicillium* 8CC2.

Factors	Effect
Average	61,2 ± 0,381881
A: soybean	−5,125 ± 0,853913^*∗*^
B: yeast extract	15,125 ± 0,853913^*∗*^
C: pH	4,625 ± 0,853913^*∗*^
D: time	9,625 ± 0,853913^*∗*^
AB	−1,375 ± 0,853913
AC	−0,875 ± 0,853913
AD	−1,375 ± 0,853913
BC	−0,125 ± 0,853913
BD	7,375 ± 0,853913^*∗*^
CD	−4,625 ± 0,853913^*∗*^

The effects that showed statistical significance (95% confidence level) are indicated by the symbol *∗*.

**Table 4:** Analysis of variance for evaluating the statistical significance of the model for biosurfactant production by *Penicillium* 8CC2 (*R*^2^ = 91.8529%).

Source	Sum of squares	Df	Mean of Squares	*F* ratio	*P* value
A: soybean oil	105,063	1	105,063	36,02	0,0093
B: yeast extract	915,063	1	915,063	313,74	0,0004
C: pH	85,5625	1	85,5625	29,34	0,0123
D: time	370,563	1	370,563	127,05	0,0015
BD	217,563	1	217,563	74,59	0,0033
CD	85,5625	1	85,5625	29,34	0,0123
Lack of fit	149,075	10	149,075	5,11	0,1030
Pure error	8,75	3	8,75		
Total	1937,2	19			

## References

[1] Raza Z. A., Rehman A., Hussain M. T. (2014). Production of rhamnolipid surfactant and its application in bioscouring of cotton fabric. *Carbohydrate Research*.

[2] Banat I. M., Franzetti A., Gandolfi I. (2010). Microbial biosurfactants production, applications and future potential. *Applied Microbiology and Biotechnology*.

[3] Pacwa-Płociniczak M., Płaza G. A., Piotrowska-Seget Z., Cameotra S. S. (2011). Environmental applications of biosurfactants: recent advances. *International Journal of Molecular Sciences*.

[4] Das P., Mukherjee S., Sen R. (2008). Improved bioavailability and biodegradation of a model polyaromatic hydrocarbon by a biosurfactant producing bacterium of marine origin. *Chemosphere*.

[5] Nitschke M., Pastore G. M. (2002). Biossurfactantes: propriedades e aplicações. *Química Nova*.

[6] Rehman A., Raza Z. A., Saif-ur-Rehman (2010). Synthesis and use of self-assembled rhamnolipid microtubules as templates for gold nanoparticles assembly to form gold microstructures. *Journal of Colloid and Interface Science*.

[7] Castiglioni G. L., Bertolin T. E., Costa J. A. (2009). Produção de biossurfactante por Aspergillus fumigatus utilizando resíduos agroindustriais como substrato. *Química Nova*.

[8] Velioglu Z., Urek R. O. (2015). Biosurfactant production by Pleurotus ostreatus in submerged and solid-state fermentation systems. *Turkish Journal of Biology*.

[9] Adrio J. L., Demain A. L. (2003). Fungal biotechnology. *International Microbiology*.

[10] Fontes G. C., Amaral P. F., Coelho M. A. (2008). Produção de biossurfactante por levedura. *Química Nova*.

[11] Pirôllo M. (2006). *Estudo da Produção de Biossurfactantes Utilizando Hidrocarbonetos*.

[12] Thavasi R., Jayalakshmi S., Balasubramanian T., Banat I. M. (2007). Biosurfactant production by Corynebacterium kutscheri from waste motor lubricant oil and peanut oil cake. *Letters in Applied Microbiology*.

[13] Pattanathu K., Rahman M., Gapke M. (2008). Production, characterisation and applications of biosurfactants - Review. *Biotechnology*.

[14] Lacaz C., Porto E., Martins J. (2001). *Martins, Microbiologia Médica: Fungos, Actinomicetos e Algas de Interesse Médico*.

[15] Barnett H. L., Hunter B. B. (1998). *IIlustrated Genera of Imperfect Fungi*.

[16] Accorsini F. R., Mutton M. J. R., Lemos E. G. M., Benincasa M. (2012). Biosurfactants production by yeasts using soybean oil and glycerol as low cost substrate. *Brazilian Journal of Microbiology*.

[17] Bodour A. A., Miller-Maier R. M. (1998). Application of a modified drop-collapse technique for surfactant quantitation and screening of biosurfactant-producing microorganisms. *Journal of Microbiological Methods*.

[18] Cameron D. R., Cooper D. G., Neufeld R. J. (1988). The mannoprotein of Saccharomyces cerevisiae is an effective bioemulsifier. *Applied and Environmental Microbiology*.

[19] Neto B. B., Scarminio I. S., Bruns R. E. (1995). *Planejamento e otimização de experimentos*.

[20] Luna-Velasco M. A., Esparza-García F., Cañízares-Villanueva R. O., Rodríguez-Vázquez R. (2007). Production and properties of a bioemulsifier synthesized by phenanthrene-degrading Penicillium sp.. *Process Biochemistry*.

[21] Camargo-de-Morais M. M., Ramos S. A., Pimentel M. C., Morais J. (2003). Production of an extracellular polysaccharide with emulsifier properties by Penicillium citrinum. *World Journal of Microbiology and Biotechnology*.

[22] Askolin S., Nakari-Setälä T., Tenkanen M. (2001). Overproduction, purification, and characterization of the Trichoderma reesei hydrophobin HFBI. *Applied Microbiology and Biotechnology*.

[23] Raza Z. A., Khan M. S., Khalid Z. M. (2007). Evaluation of distant carbon sources in biosurfactant production by a gamma ray-induced Pseudomonas putida mutant. *Process Biochemistry*.

[24] Kim H.-S., Yoon B.-D., Lee C.-H. (1997). Production and properties of a lipopeptide biosurfactant from *Bacillus subtilis* C9. *Journal of Fermentation and Bioengineering*.

[25] Makkar R. S., Cameotra S. S. (1998). Production of biosurfactant at mesophilic and thermophilic conditions by a strain of Bacillus subtilis. *Journal of Industrial Microbiology and Biotechnology*.

[26] Banat I. M., Desai D. J. (1997). Microbial production of surfactants and their commercial potential. *Microbiology and Molecular Biology Reviews*.

[27] Decesaro A., Rigon M. R., Thomé A., Colla L. M. (2013). Produção de biossurfactantes por microrganismos isolados de solo contaminado com óleo diesel. *Química Nova*.

[28] Kim H.-S., Jeon J.-W., Kim B.-H., Ahn C.-Y., Oh H.-M., Yoon B.-D. (2006). Extracellular production of a glycolipid biosurfactant, mannosylerythritol lipid, by Candida sp. SY16 using fed-batch fermentation. *Applied Microbiology and Biotechnology*.

[29] Castiglioni G. L., Stanescu G., Rocha L. A. O., Costa J. A. V. (2014). Acta scientiarum analytical modeling and numerical optimization of the biosurfactants production in solid-state fermentation by aspergillus fumigatus. *Acta Scientiarum*.

[30] Kiran G. S., Hema T. A., Gandhimathi R. (2009). Optimization and production of a biosurfactant from the sponge-associated marine fungus Aspergillus ustus MSF3. *Colloids and Surfaces B: Biointerfaces*.

[31] Acioly L. M., Silveira A., Anjos M., Mendez-Vilas A. (2012). Biosurfactant production by Mucor circinelloides using apple peel, vegetable oil and corn steep liquor as substrate. *Microbes in Applied Research–Currents Advances and Challenges*.

[32] Monteiro A. S., Bonfim M. R. Q., Domingues V. S. (2010). Identification and characterization of bioemulsifier-producing yeasts isolated from effluents of a dairy industry. *Bioresource Technology*.

[33] Khopade A., Ren B., Liu X.-Y., Mahadik K., Zhang L., Kokare C. (2012). Production and characterization of biosurfactant from marine Streptomyces species B3. *Journal of Colloid and Interface Science*.

[34] Mullingan C., Cooper D., Neufeld R. (1984). Selection of microbes producing biosurfactants in media without hydrocarbons. *Journal of Fermentation Technology*.

