# Erratum to “Production of Biosurfactants by Soil Fungi Isolated from the Amazon Forest”

**DOI:** 10.1155/2018/1586859

**Published:** 2018-09-06

**Authors:** Hellen Holanda Sena, Michele Alves Sanches, Diego Fernando Silva Rocha, Walter Oliva Pinto Filho Segundo, Érica Simplício de Souza, João Vicente Braga de Souza

**Affiliations:** ^1^Post-Graduate Program in Pharmaceutical Sciences, Federal University of Amazonas (UFAM), R. Alexandre Amorim 330, 69010-300 Manaus, AM, Brazil; ^2^Department of Medical Microbiology, National Institute of Amazonian Research (INPA), Av. André Araújo 2936, 69080-971 Manaus, AM, Brazil; ^3^Superior School of Technology, Amazonas State University (UEA), Av. Darcy Vargas 1200, 69050-020 Manaus, AM, Brazil

In the article titled “Production of Biosurfactants by Soil Fungi Isolated from the Amazon Forest” [1], the Acknowledgements section was missing from the published article due to a production error and should be added as follows.
